# Preventing LVAD implantation by early short-term mechanical support and prolonged inodilator therapy: A case series with acute refractory cardiogenic shock treated with veno-arterial extracorporeal membrane oxygenation and optimised medical strategy

**DOI:** 10.1007/s12471-013-0509-5

**Published:** 2014-01-15

**Authors:** J. J. Brugts, O. Manintveld, A. Constantinescu, D. W. Donker, R. J. van Thiel, K. Nieman, L. S. D. Jewbali, F. Zijlstra, K. Caliskan

**Affiliations:** 1 Department of Cardiology, Erasmus MC Thoraxcenter, ’s Gravendijkwal 230, 3015CE Rotterdam, the Netherlands; 2 Department of Intensive Cardiac Care Unit, Erasmus MC Thoraxcenter, Rotterdam, the Netherlands; 3 Department of Cardiology, Maastricht University Medical Center, Maastricht, the Netherlands

**Keywords:** Heart failure, Cardiogenic shock, LVAD, Heart transplantation, Inodilator, INTERMACS

## Abstract

Cardiogenic shock continues to be a life-threatening condition carrying a high mortality and morbidity, where the prognosis remains poor despite intensive modern treatment modalities. In recent years, mainly technical improvements have led to a more widespread use of short- and long-term mechanical circulatory support, such as veno-arterial extracorporeal membrane oxygenation (VA-ECMO) and left ventricular assist devices (LVADs). Currently, LVADs are indispensable as ‘bridge’ to cardiac recovery, heart transplantation (HTX), and/or as destination therapy Importantly, both LVADs and HTX put a vast burden on financial resources, besides significant short- and long-term risks of morbidity and mortality. These considerations underscore the importance of optimal timing and appropriate patient selection for LVAD therapy, avoiding as much as possible an unfortunate and costly clinical path. In this report, we present a series of three cases with acute refractory cardiogenic shock (‘crash and burn’, INTERMACS profile 1) successfully treated by ECMO and early optimal medical therapy preventing a certain path towards LVAD and/or HTX, for which they were initially referred. This conservative approach in INTERMACS profile one patients warrants very early introduction of adequate medical heart failure therapy under the umbrella of a combination of short-term mechanical circulatory and inotropic support by phosphodiesterase inhibitors. Therefore, this novel combined medical-mechanical approach could have important clinical implications for this extremely challenging patient category, as it may avoid an unnecessary and costly clinical path towards LVAD and/or heart transplantation.

## Introduction

Cardiogenic shock continues to be a highly dangerous condition carrying considerable risk of mortality and morbidity despite all currently available treatment modalities. In clinical practice, urgent resuscitation by short-term mechanical support is often the only remaining therapeutic option to prevent a certain death [1]. Among the available devices, veno-arterial extra-corporeal membrane oxygenation (ECMO) has been suggested to be the most useful initial step for urgent stabilisation in severe, refractory cardiogenic shock [1]. More long-term support and effective ventricular unloading is best achieved by surgical implantation of a left ventricular assist device (LVAD) [1]. Theoretically, high urgency heart transplantation (HTX) poses an alternative strategy in the acute setting, but is hampered by scarceness of donor organs and is therefore virtually impossible. Currently, the use of LVADs is increasing rapidly in popularity as ‘bridge-to-recovery’, ‘bridge-to-transplantation’, or ‘destination therapy’ [1, 2], in the absence of generally accepted practice guidelines to aid in optimal patient selection and cost-effectiveness. The rationale of LVAD implantation in patients with acute refractory cardiogenic shock often poses an ultimate therapeutic step, but is well known to carry a considerable morbidity and mortality. Recently, results of the INTERMACS registry have clearly shown that the postoperative outcome of advanced heart failure patients undergoing ventricular assist device implantation is strongly influenced by their preoperative INTERMACS profile [3]. INTERMACS profile one patients are defined by cardiogenic shock with persisting haemodynamic instability in spite of increasing doses of inotropes and IABP support with critical hypoperfusion of target organs [3]. In that sense, it is evident that patients suffering severe cardiogenic shock, i.e. defined as INTERMACS profile 1, generally had a deleterious outcome. The authors argue that their results call for a change in policies related to the management of heart transplant and LVAD candidates in this specific patient category due to their deleterious outcome [3].

Here, we present an alternative therapeutic strategy, exemplified by a series of three cases presenting with acute, severe and refractory cardiogenic shock and successfully treated by early veno-arterial ECMO (Table 1). The latter was combined with optimised medical therapy, i.e. early but gradual titration of low-dose beta-blocker therapy under the ‘umbrella’ of phosphodiesterase inhibitors, for a prolonged period of time. All three patients survived the initial critical phase of severe cardiogenic shock (‘crash and burn’, INTERMACS profile 1) and recovered to such a degree that there was no need for other definite and irreversible therapeutic solutions such as LVAD implantation or heart transplantation.

**Table 1:** Patient characteristics

	Patient A	Patient B	Patient C
Age	28 years	25 years	50 years
Gender	Male	Female	Male
Medical history	Alcohol and drug abuse	None	Depression
Complaints	Progressive fatigue and shortness of breath	Muscle pain, nausea, vomiting and dizziness since 1 week	Muscle pain, subfebrile temperatures, fatigue and stomach pain since 1 week
Admission with	Acute severe heart failure, cardiogenic shock	Acute severe heart failure, cardiogenic shock	Acute severe heart failure, cardiogenic shock
Echocardiography	Severely impaired systolic LV function with dilated left ventricle with mild mitral insufficiency.	Severely impaired systolic LV and RV function, thrombus in right ventricle	Severely impaired systolic LV function, dilated cardiomyopathy, large LV thrombus
Additional studies	Laboratory: lactate acidosis and multi-organ failure (ATN, shock liver) MRI showed dilated cardiomyopathy without any signs of acute myocarditis	Laboratory: lactate acidosis and multi-organ failure (ATN, shock liver) Virology: PCR positive for parvo B19 virus Endomyocardial biopsy: parvo B19 virus.	Laboratory: lactate acidosis and multi-organ failure (ATN, shock liver) Endomyocardial biopsy negative
Diagnosis	Refractory cardiogenic shock due to toxic cardiomyopathy (alcohol and amphetamine)	Refractory cardiogenic shock based on parvo B19 viral myocarditis	Refractory cardiogenic shock based on de novo dilated CMP e.c.i.
Initial approach	High-dose positive inotropes, CVVH and IABP	High-dose positive inotropes and IABP	High-dose positive inotropes, and IABP
Therapeutic approach	VA-ECMO short term mechanical support Enoximone 1 mg/kg/min intravenously with early introduction of low-dose beta blocker/ACEi	VA-ECMO short-term mechanical support Enoximone 1 mg/kg/min intravenously with early introduction of low-dose beta blocker/ACEi	VA-ECMO short-term mechanical support Enoximone 1 mg/kg/minintravenously with early introduction of low-dose beta blocker/ACEi
VA ECMO duration	11 days	7 days	10 days
Complications	Episode of thrombocytopenia and HIT	3rd degree AV block, DDD pacemaker	None
Medication at discharge	Acenocoumarol; Bisoprolol 10 mg qd; Ramipril 3.75 bid; Furosemide 20 mg qd; Esomeprazole 40 mg bid; Quetiapine 25 mg bid.	Ramipril 5 mg bid; Metoprolol Succinate 100 mg bid; Furosemide 40 mg qd; Ferrofumarate 200 mg tid; Esomeprazole 40 mg qd	Acenocoumarol; Ramipril 7.5 mg bid; Metoprolol Succinate 50 mg bid; Digoxin 0.125 mg qd; Amiodarone 200 mg qd; Furosemide 40 mg qd; Spironolactone 12.5 mg qd; Esomeprazole 40 mg qd
Echocardiogram at short-term follow-up	4 weeks after admission: moderately impaired systolic LV function, mild mitral insufficiency.	5 weeks after admission: mildly impaired systolic LV and RV function, no signs of thrombus or valve insufficiency	6 weeks after admission: mildly impaired systolic LV and RV function, no signs of thrombus or significant valve insufficiency
Long-term follow-up	At 1.5 years follow-up, he is asymptomatic, NYHA class I. Echo: estimated EF 35–40 %	At 3.5 years follow-up, she is asymptomatic, NYHA class I. Echo: estimated EF 40–45 %	At 2.5 years follow-up, he is asymptomatic, NYHA class I. Echo: estimated EF 45 %

*ACEi* angiotensin converting enzyme inhibitors; *AV* atrio-ventricular; *ATN* acute tubular necrosis; *CMP* cardiomyopathy; *CVVH* continuous veno-venous hemofiltration; *e.c.i.* e causa ignota; *EF* ejection fraction, *IABP* intra-aortic balloon pump; *HF* heart failure; *HIT* heparin-induced thrombocytopenia; *LV* left ventricular; *RV* right ventricular; *MRI* magnetic resonance imaging; *NYHA* New York Heart Association; *VA-ECMO* veno-arterial extra-corporeal membraneous oxygenation

## Case series

### Patient A

**Fig. 1:**
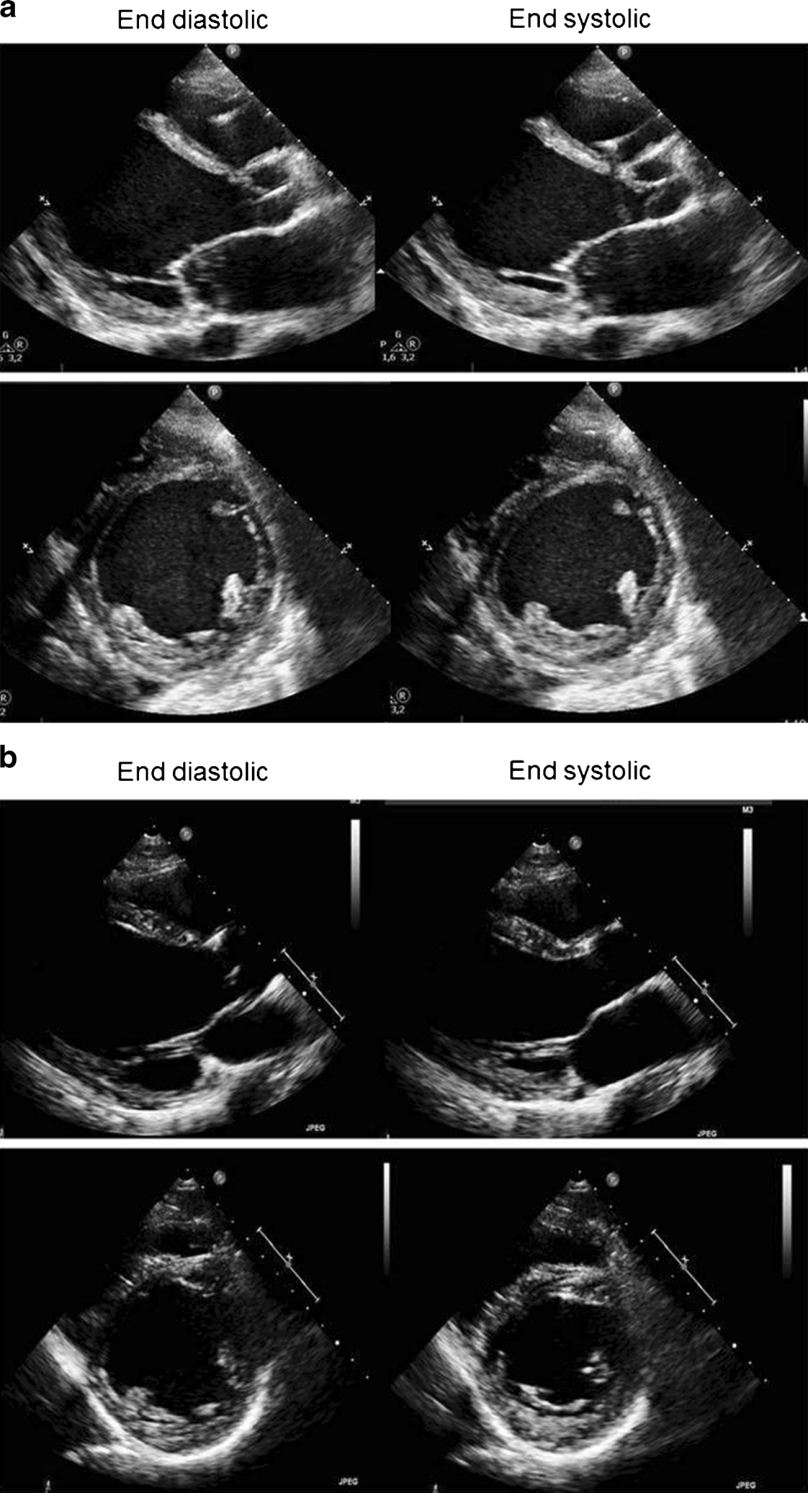
**a** Initial phase (week 1). Transthoracic echocardiographic images representative of case 1; diastolic (*left*) and systolic (*right*) still frames from parasternal long axis, short axis views. Side box. LVEDD 68 mm. LVESD 62 mm. Estimated LVEF 10 %. **b** After ECMO and heart failure medical treatment (6 months). Transthoracic echocardiographic images representative of case 1; diastolic (*left*) and systolic (*right*) still frames from parasternal long axis, short axis views. Side box. LVEDD 65 mm. LVESD 44 mm. Estimated LVEF 35–40 %

**Fig. 2:**
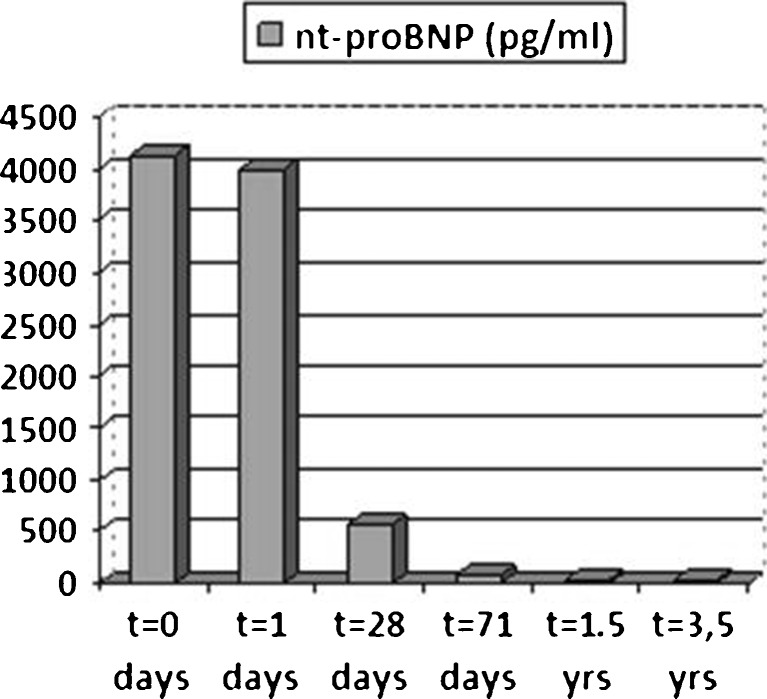
Serial NTproBNP levels in case 2 from presentation with cardiogenic shock to ECMO introduction at day 2 (total VA-ECMO time 7 days), including enoximone (total time 16 days) and heart failure medication with rapidly declining NTproBNP levels in line with clinical recovery (discharge in good condition after 36 days)

A 28-year-old male without any cardiovascular history but known alcohol and drug (amphetamine) abuse, was admitted to our Intensive Cardiac Care Unit with acute, severe and refractory cardiogenic shock (Table 1). Laboratory tests revealed concomitant multi-organ failure: acute kidney injury requiring renal replacement therapy, elevated liver enzymes and lactate levels. The electrocardiogram showed a normally conducted sinus tachycardia (138 beats/min), and signs of left atrial dilatation, but no ischaemia. On echocardiography, the left ventricle was extensively dilated (left ventricular (LV) end-diastolic diameter 68 mm) exhibiting a severely impaired LV contractility and mild mitral regurgitation (Fig. 1a). Coronary angiography revealed no significant lesions and acute myocarditis and fibrosis were excluded by cardiac magnetic resonance imaging. Initial treatment with inotropics and intra-aortic balloon pump (IABP) was insufficient, necessitating veno-arterial (VA) ECMO to stabilise the patient. The patient had an INTERMACS profile 1 as defined [3]. Given the limited maximal ECMO support duration of a few weeks, urgent HTX or ‘bridge-to-HTX’ LVAD therapy was discussed early but considered contraindicated due to expected noncompliance related to active alcohol and drug abuse. As a possible alternative, we decided to prolong the VA-ECMO therapy as bridge-to-recovery and we started by introducing regular heart failure therapy in a very early stage. This regimen included an ACE inhibitor (ramipril) and beta blockade (bisoprolol) at the lowest possible dosages, importantly under the umbrella of phosphodiesterase inhibition (enoximone). Under this regimen, the patient survived this initial critical phase (‘crash to certain death’) while his cardiac function and multi-organ failure gradually improved. After 11 days of VA-ECMO, the patient could be successfully weaned from mechanical ventilation and ECMO. During the following weeks, the HF treatment was further intensified in dosage and the patient recovered uneventfully. LV function recovered to a moderately impaired LV function in 4 weeks. At long-term follow-up (18 months), the patient remained asymptomatic and his LV function remained stable (estimated EF 35–40 %) on standard heart failure medication including bisoprolol, ramipril, digoxin and spironolactone (Figs. 1b and 2). Thereafter, the patient complied irregularly with his visits at our outpatient heart failure clinic, in the end withdrawing fully from further visits.

### Patient B

A 25-year-old female with no history of cardiovascular disease was admitted with severe cardiogenic shock and decompensated heart failure after a week of fever, myalgia, nausea and vomiting (Table 1). Blood results demonstrated multi-organ failure and elevated lactate levels. Echocardiography demonstrated biventricular dilatation with severely impaired contractility and presence of a right ventricular (RV) intracavitary thrombus. Cardiogenic shock was refractory to optimal medical treatment (high-dose inotropes) and her clinical condition deteriorated rapidly. The patient had an INTERMACS profile 1 as defined [3]. Based on the clinical presentation, acute myocarditis was suspected as being a potentially reversible underlying cause. Therefore circulatory support using VA-ECMO was initiated. Subsequently, the diagnosis of acute myocarditis could be confirmed by RV endomyocardial biopsies showing extensive lymphocytic myocardial infiltration and evidence of recent Influenza A and parvo virus B19 infection. During VA-ECMO therapy, alongside low-dose enoximone, we gradually introduced bisoprolol and ramipril at low dosages. The patient improved clinically and her renal function and liver enzymes normalised. After 7 days of mechanical support, ECMO could be weaned. The clinical course was complicated by 3rd degree AV block necessitating DDD-pacemaker implantation. The patient’s further recovery was uneventful under standard heart failure therapy. Enoximone could be stopped after 16 days, and she was discharged after 36 days of hospital admission. Regular follow-up echocardiograms showed an improvement in cardiac function. At 3.5 years of follow-up, she remains fully asymptomatic with only mildly depressed LV function with estimated ejection fraction (EF) of 40–45 %, normal LV dimensions (LVEDD 48 mm) and normal LV filling pressures (E/E’ 8).

### Patient C

A 50-year-old male with no relevant medical history presented to a referring hospital with severe left-sided heart failure with signs of dilated cardiomyopathy on echocardiography (Table 1). He complained of myalgia, fatigue and subfebrile temperature in the past week. Electrocardiogram revealed a sinus tachycardia of 100 beats/min, with signs of left atrial dilatation and LV strain pattern. Echocardiography showed a severely impaired LV function and dilated left ventricle with apical LV thrombus. Due to progressive cardiogenic decline with high suspicion of fulminant myocarditis, a VA-ECMO was implanted alongside high dosages of positive inotropics and IABP as well as high-dose corticosteroids, immunoglobulins and levosimendan. The patient had an INTERMACS profile 1 as defined [3]. Because of possible candidacy for an urgent LVAD implantation as a bridge-to-HTx, the patient was referred to our tertiary centre. After transfer to our centre, the IABP could be removed and inotropes were switched from dobutamine to enoximone during VA-ECMO support. At this stage, endomyocardial biopsy did not show active myocarditis nor any other diagnostic clues. Therefore, the immunosuppressants were stopped. Again, we introduced low-dose metoprolol and ramipril while continuing enoximone therapy and VA-ECMO circulatory support. After 10 days of VA-ECMO support, the patient could be successfully weaned from mechanical support. With continuing low-dose enoximone his heart failure therapy was further optimised by increasing the dose of metoprolol and ramipril. After 2 weeks of admission the patient improved further; also the enoximone could be stopped and standard heart failure therapy was titrated. The patient fully recovered and was transferred in a stable clinical condition to the referring hospital. Regular follow-up echocardiograms demonstrated a gradual improvement in cardiac function to moderately impaired LV function without significant valvular insufficiency and the apical thrombus had vanished. After 2.5 years of follow-up the patient remained asymptomatic with mildly impaired LV function with an estimated of EF 45 %.

## Discussion

Here, we present a series of cases with acute, severe and refractory cardiogenic shock, successfully treated with ‘short-term’ ECMO support and optimised medical therapy preventing a certain path towards LVAD and/or heart transplantation. Importantly, this more conservative approach in INTERMACS profile 1 incorporates early introduction of heart failure therapy, already during ECMO, using phosphodiesterase inhibition as an ‘umbrella’ to allow early initiation of oral heart failure therapy including beta blockers and angiotensin enzyme inhibitors.

We hypothesised that the very early initiation of medical-mechanical support and introduction of heart failure medication (under the umbrella of enoximone) provides the *momentum* for these patients to survive the first critical phase (crash to certain death) and afterwards continue in the upward line of clinical recovery, thereby preventing the path towards LVAD or HTX.

Cardiogenic shock remains a highly dangerous condition with a high risk of mortality and morbidity despite extensive current medical and mechanical support [1–3]. With increasing availability of short mechanical circulatory support and long-term solutions such as left ventricular assist devices (LVADs), therapeutic options in cardiogenic shock patients are increasingly broadened [3–6]. ECMO is reported to be successful as a bridge-to-recovery in out-of-hospital patients presenting with severe cardiogenic shock [4–6]. With current technological improvements, ECMO has developed into a lightweight portable and reliable device which, in experienced hands, is easily implanted percutaneously via the femoral vessels in 15 min. It is therefore more applicable in acute settings (cardiogenic shock) and the best available short-term mechanical support device which can be used on a temporary basis (removed easily). As a long-term solution, the path of LVAD and/or HTX still has major drawbacks with huge impact on financial resources [3–6]. Additionally, there is an extreme shortage of suitable heart donors, and a significant morbidity and mortality with LVAD implantation. This warrants some precautions and discussion on the best timing and right patient selection before turning into an irreversible pathway. Therefore, we question whether LVADs are the best choice in all and we advocate that we keep trying to think of a way to divert the path of LVAD or HTX waiting list in these patient groups, especially in the first weeks to evaluate whether the patient can rather recover with ECMO bridging. Our case series shows that short-term mechanical support is suitable as bridge-to-decision in order to identify the right candidates for LVAD or HTX. Current ESC guidelines on the diagnosis and treatment of heart failure are brief on the topic of mechanical assist devices due to the lack of evidence and clinical data, and clinical expert opinion is still important, which makes our case series clinically relevant as well as the discussion it provokes, aimed to improve patient care in this vulnerable subset of patients.

In this case series, we discuss an alternative approach in selected patients with severe cardiogenic shock to use a VA-ECMO as bridge-to-recovery, enhanced by introducing very early heart failure medication, including ACE inhibitors and beta blockers using enoximone and ECMO. Our approach of using VA-ECMO for a prolonged period of time under the umbrella of enoximone therapy is new and could be a way to prevent the bridge-to-destination or bridge-to-transplantation paths. The current approach has worked for several of our patients with great success, as presented in our case series. However, patient numbers (and experience) in this category will remain small and dependent largely on clinical experience. We believe that –in our centre- this approach was partly feasible due to the unique cooperation of cardiac intensive care specialists as well as heart failure specialists in a combined intensive care unit and cardiac care unit.

The proposed period of bridging with VA-ECMO and very early introduction of heart failure therapy (including beta blockers) under the umbrella of enoximone, can give the needed time to evaluate the clinical course of the patient’s illness and select the appropriate patients to either await clinical recovery or proceed to LVAD. After stabilisation, enoximone provided the necessary back-up to introduce B1-selective beta blockers as these agents are independent of beta-receptor signalling and beta receptors in contrast to dobutamine [7, 8]. The site of action of phosphodiesterase inhibitors is beyond the beta-adrenergic receptor and the two agents have additive effects [7, 8]. We argue that the early introduction of beta-blocker therapy is essential in the recovery phase. A gradual increase of the beta-blocker dose under the safety of enoximone and VA-ECMO will avert the feared haemodynamic compromise [7, 8] and save enormous time in patient recovery (which depends on introduction of heart failure therapy). Still, the early introduction of beta-blocker therapy seems contra-intuitive and is challenged by other experts [7, 8].

In all three patients, cardiogenic shock and multi-organ failure persisted despite conventional support which was the reason to expand treatment with VA-ECMO rapidly as an option for short-term (temporary) mechanical support. The choice for VA-ECMO depends on experience and device availability in the centre. VA-ECMO can be inserted – percutaneously – rapidly and buy time. It is essential to take this decision as early as possible on the first day, before multi-organ failure has advanced to such a degree that VA-ECMO is also doomed to fail. Realistically, some patients will cope (as presented in the current cases), but others will not and still need LVAD or heart transplantation despite all efforts [9]. VA-ECMO can be removed and is a temporary device. The INTERMACS study taught us that patients in cardiogenic shock have a deleterious outcome with urgent LVAD implantation and these results call for a change in management in this particular patient category, Therefore, our conservative regimen targets INTERMACS profile 1 patients and may provide us with the valuable time to evaluate this patient category for proper selection. In our opinion this should be attempted, as an ‘LVAD for all’ approach is detrimental to these patients with INTERMACS profile 1 [3] as well as not manageable on a large scale. While searching for alternative treatment, we realise that our regimen requires a high dedication and patience from the whole clinical team. The current regimen should be tested in larger studies which may provide more insight into the best applicable patients as well as the effect of the underlying cause of acute heart failure on decision-making.

In conclusion, we advocate an initial *conservative approach* in INTERMACS profile 1 patients by combining short-term mechanical circulatory support with early introduction of medical heart failure therapy under the umbrella of phosphodiesterase inhibitors, which could be a successful way to treat refractory cardiogenic shock patients. This novel approach may have clinical implications as it shows that the clinical path towards LVAD and/or heart transplantation can be diverted in some, which makes the discussion on applicable patients for LVADs even more challenging and efforts to reduce unnecessary LVAD implantation even more warranted.
